# SLAP lesions, An Opinion Piece

**DOI:** 10.2174/1874325001812010342

**Published:** 2018-07-31

**Authors:** Cecilie P. Schrøder

**Affiliations:** Department Lovisenberg Diaconal Hospital, Oslo, Norway

**Keywords:** SLAP repair, Biceps tenodesis, Labral repair, Surgery, SLAP lesion, Biceps tenotomy

## Abstract

SLAP lesions were first classified by Snyder in 1990. Results of treatment have been controversial without clear consensus. All have agreed that prospective studies would be useful. We conducted such a study between 2008 to 2114 that randomized treatment between sham surgery, biceps tenodesis and labral repair. No significant differences in results between the groups were found. Crossover between groups was only possible from the sham surgery group and this may introduce some degree of bias. However, the six month outcomes between all three groups before any crossover were statistically identical. Our results also do not favor biceps tenodesis *versus* SLAP repair when surgery is performed. Based on these results we have narrowed our indications for SLAP lesion surgery. We still treat some SLAP lesions surgically and individualize our treatment in each such cases. Most SLAP lesion patients, however, are ultimately treated non-operatively.

## 1 BACKGROUND

Discussion of the clinical importance and treatment of type II SLAP lesions has a history spanning more than 25 years. The classification by Snyder *et.al*. [1] in 1990 initiated a decade of attempts to find the optimal surgical treatment of this entity, and suture anchor repair has been the dominant method. Retrospective, level IV studies showed promising results, but systematic reviews [2, 3] reached more nuanced conclusions

Gorantla *et al.* [2] concluded in their review from 2010 that the results after SLAP repair were excellent for individuals not involved in throwing or overhead sports but they were less predictable for those engaged in such sports. The authors found no level I or II evidence for the outcome of SLAP repair and they recommended prospective studies. A review by Huri *et al.* [3] in 2014 concludes that the evaluation and treatment of SLAP lesions continues to be controversial. They state that the results after repair have been shown to be less successful than initially reported, and that dissatisfaction with the results has led to an increased use of biceps tenotomy or tenodesis as the initial treatment, especially in older individuals. They also conclude that the role of biceps tenodesis or tenotomy in the overhead athlete is controversial, and that the use of SLAP repair in this population remains uncertain.

In 2012, our group published a 5-year follow-up of isolated SLAP repairs in a prospective study [4]. At that point, we were only aware of one other prospective study, the study by Brockmeier *et al*. [5] from 2009. The results from these two studies are almost identical, with 88% and 87% success rates, respectively. Brockmeier reported 74% return to prior level of competition and postoperative refractory stiffness in 4/47 patients. We found stiffness in 13% of patients and the only athletes that did not return to their prior level of activity were team handball players. We concluded that the long-term outcomes after SLAP repairs were good and independent of age. The non-randomized study design and lack of a control group were important limitations, and postoperative rehabilitation, patient characteristics and the natural course might have contributed to the outcomes. We stated that to obtain Level I or II evidence, randomized studies designed to compare SLAP repair, biceps tenodesis and non-operative treatment were needed.

Based on this background, we conducted a clinical trial [6] from 2008 to 2014, in which 118 patients with a mean age of 40 years were randomized to labral repair, biceps tenodesis or sham (placebo) surgery. Study inclusion occurred during arthroscopy once an isolated type II SLAP was verified. The patients had regular clinical follow-up, and a final 2-year follow-up. The primary outcome measures were Rowe score and Western Ontario Shoulder Index (WOSI) administered 6 and 24 months after surgery. Patient satisfaction was assessed separately using a self-reported questionnaire with response alternatives of poor, fair, good and excellent. The secondary outcome measures were Oxford Instability Shoulder Score (OISS) and EQ-VAS. All scores were validated for use with patients with SLAP lesions [7-9]. The patients, the treating physiotherapists/manual therapists and the person collecting and analyzing the data were blinded to the study group assignment.

## 2 RESULTS

The results showed significant improvement in both subjective and objective scores for all three groups, but surprisingly, no significant group differences in the intention to treat analysis. The Rowe and WOSI scores at two years were 86.5 and 436, respectively, for the sham group; 86.8 and 455 for tenodesis; and 86.3 and 334 for labral repair. Patient satisfaction was 85% excellent/good in the sham group, 89% in the tenodesis group and 83% in the labral repair group. Fourteen patients from the sham group crossed over after 6 months.

Both intention- to- treat and per- protocol analysis were performed and we found no statistical differences between the three groups. We are aware of the discussion regarding possible bias and underestimation of potential benefit of a treatment using intention to treat analysis in studies with only one-way cross-over. In such studies, only the patients in the sham-group have the possibility to cross over, and it is claimed that a patient with more severe symptoms is more likely to cross over from non-operative treatment than a patient with less severe symptom. Thus, comparing these two groups of patients may introduce a bias.

We cannot claim that this is not the case in our study where 14/39 (35.9%) patients in the sham group were reoperated. But, the mean Rowe and WOSI scores at 6 months (before cross-over) in these 14 patients, were not significantly different from the 14 patients with the lowest scores in the labral repair and biceps tenodesis groups. This comparison suggests that the threshold for reoperation was lower in the sham group and we cannot rule out that the higher rate of reoperation in the sham group was related to unblinding rather than to differences in treatment in treatment failure. At final follow up, the mean Rowe and WOSI scores in this cross-over group were significantly lower than for the three original groups. This is a small group and meaningful analysis was not possible, but it seems as if some of the effect may have been lost in the mean. One might interpret the lower mean scores as indicating that the cross-over group had no effect from the operative treatment, but eight of the 14 cross-over patients had a Rowe score above 80 and rated their shoulder as good or excellent. Thus, we may have underestimated the potential benefit of labral repair and biceps tenodesis. Never the less, 64.1% of the patients in the sham group did not require any additional treatment and it is reasonable to think that a proportion of both the patients in the labral repair and the biceps tenodesis group could have managed well non-operatively as well.

The critics state that mean age of our patients was too high and that only young active patients with a traumatic SLAP should be treated. When we initiated this study, we wanted to describe the situation in our daily practice. There were no high-level studies and only a few prospective studies, and the results were divergent concerning both whether the age of the patient mattered and the degree of return to prior overhead activity.

As isolated SLAP lesions are quite rare, it took us 6.5 years to include our 118 patients. During that period, reports of a large increase in the number of SLAP repairs being performed, along with reports of complications, were published [10-13]. Many experienced shoulder surgeons stated that they had already narrowed their indication for such a repair. Later reports [14, 15] of a decrease in both the number of repairs and the age of the patient undergoing these operations, and the increasing use of biceps tenodesis as an alternative method, shows that the orthopedic community has responded to the emerging information about SLAP repairs. However, we still lack high-level evidence of how to treat these patients.

Our results extend previous reports [11, 12] of possible overtreatment of SLAP lesions and indicate a need to narrow indications. Patient age is debated and authors advocate that SLAP repair should be reserved for the young and active patient. We found no significant differences in function, patient satisfaction or complications by age in this study, but the groups are too small to perform subgroup analysis and identify factors associated with failures. In addition, our group is a mixed population of physically active adults, but relatively few overhead athletes. Thus, further studies are needed to establish the best treatment for the young active patient. The present study does not support either labral repair or biceps tenodesis in this population, as we found no significant differences between treatments on any outcome. Considering the lack of high-quality trials in this field, the results of this study should be interpreted with caution. It is also important to note that although physiotherapy might have contributed to the improvements observed across all groups, the impact of placebo, the natural course and regression to the mean should not be underestimated.

Based on our current knowledge, our indication for SLAP repairs is significantly narrowed, but we still perform them in selected cases. We inform our patients about the possibility of a good result with adequate non-operative treatment and follow our study protocol with regard to rehabilitation. The majority of our patients follow this regime. They are followed up with a clinical examination at three and six months, and dependent on their progress, a decision regarding operative treatment is made. A young overhead athlete, such as a team handball player or a gymnast is treated with a labral repair plus a tenodesis if non-operative treatment fails. Other young physically active patients not engaged in overhead sports, might receive a labral repair, but if there are significant biceps groove symptoms, we perform a tenodesis. Older patients (over age 40) typically receive a bicepstenotomy or tenodesis depending on physical demands, body habitus and preference.

## CONCLUSION

With respect for the clinical knowledge Snyder and co-workers have in this field, we fully agree with their statement [16] of genuine respect for the SLAP tear, and that each case of a suspected SLAP lesion must be evaluated on its own merits. Nevertheless, we believe that many of these patients can be managed non-operatively.

## References

[1] Snyder S.J., Karzel R.P., Del Pizzo W., Ferkel R.D., Friedman M.J. (1990). SLAP lesions of the shoulder.. Arthroscopy.

[2] Gorantla K., Gill C., Wright R.W. (2010). The outcome of type II SLAP repair: A systematic review.. Arthroscopy.

[3] Huri G., Hyun Y.S., Garbis N.G., McFarland E.G. (2014). Treatment of superior labrum anterior posterior lesions: A literature review.. Acta Orthop. Traumatol. Turc..

[4] Schrøder C.P., Skare O., Gjengedal E., Uppheim G., Reikerås O., Brox J.I. (2012). Long-term results after SLAP repair: A 5-year follow-up study of 107 patients with comparison of patients aged over and under 40 years.. Arthroscopy.

[5] Brockmeier S.F., Voos J.E., Williams R.J., Altchek D.W., Cordasco F.A., Allen A.A., Hospital for Special Surgery Sports Medicine and Shoulder Service (2009). Outcomes after arthroscopic repair of type-II SLAP lesions.. J. Bone Joint Surg. Am..

[6] Schrøder C.P., Skare Ø., Reikerås O., Mowinckel P., Brox J.I. (2017). Sham surgery *versus* labral repair or biceps tenodesis for type II SLAP lesions of the shoulder: A three-armed randomised clinical trial.. Br. J. Sports Med..

[7] Skare Ø., Schrøder C.P., Mowinckel P., Reikerås O., Brox J.I. (2011). Reliability, agreement and validity of the 1988 version of the Rowe Score.. J. Shoulder Elbow Surg..

[8] Skare Ø., Mowinckel P., Schrøder C.P., Liavaag S., Reikerås O., Brox J.I. (2014). Responsiveness of outcome measures in patients with superior labral anterior and posterior lesions.. Shoulder Elbow.

[9] Skare Ø., Liavaag S., Reikerås O., Mowinckel P., Brox J.I. (2013). Evaluation of Oxford instability shoulder score, Western Ontario shoulder instability index and Euroqol in patients with SLAP (superior labral anterior posterior) lesions or recurrent anterior dislocations of the shoulder.. BMC Res. Notes.

[10] Katz L.M., Hsu S., Miller S.L., Richmond J.C., Khetia E., Kohli N., Curtis A.S. (2009). Poor outcomes after SLAP repair: descriptive analysis and prognosis.. Arthroscopy.

[11] Vogel L.A., Moen T.C., Macaulay A.A., Arons R.R., Cadet E.R., Ahmad C.S., Levine W.N. (2014). Superior labrum anterior-to-posterior repair incidence: A longitudinal investigation of community and academic databases.. J. Shoulder Elbow Surg..

[12] Weber S.C., Martin D.F., Seiler J.G., Harrast J.J. (2012). Superior labrum anterior and posterior lesions of the shoulder: Incidence rates, complications, and outcomes as reported by American Board of Orthopedic Surgery. Part II candidates.. Am. J. Sports Med..

[13] Boileau P., Parratte S., Chuinard C., Roussanne Y., Shia D., Bicknell R. (2009). Arthroscopic treatment of isolated type II SLAP lesions: Biceps tenodesis as an alternative to reinsertion.. Am. J. Sports Med..

[14] Erickson B.J., Jain A., Abrams G.D., Nicholson G.P., Cole B.J., Romeo A.A., Verma N.N. (2016). SLAP lesions: trends in treatment.. Arthroscopy.

[15] Patterson B.M., Creighton R.A., Spang J.T., Roberson J.R., Kamath G.V. (2014). Surgical trends in the treatment of superior labrum anterior and posterior lesions of the shoulder: Analysis of data from the American Board of Orthopaedic Surgery Certification Examination Database.. Am. J. Sports Med..

[16] Burns J.P., Bahk M., Snyder S.J. (2011). Superior labral tears: Repair versus biceps tenodesis.. J. Shoulder Elbow Surg..

